# Adverse childhood experiences are associated with spontaneous preterm birth: a case–control study

**DOI:** 10.1186/s12916-015-0353-0

**Published:** 2015-06-11

**Authors:** Inge Christiaens, Kathleen Hegadoren, David M Olson

**Affiliations:** Department of Obstetrics and Gynaecology, Newcastle University, Newcastle upon Tyne, NE2 4HH UK; Faculty of Nursing, University of Alberta, Edmonton, Alberta T6G 2S2 Canada; Departments of Obstetrics and Gynecology, Pediatrics and Physiology, University of Alberta, Edmonton, Alberta T6G 2S2 Canada

**Keywords:** Adverse childhood experiences, Maternal stress, Preterm birth

## Abstract

**Background:**

More than 1 in 10 infants are born prematurely worldwide, making preterm birth the leading cause of neonatal mortality and morbidity. Chronic maternal stress is increasingly recognized as one of the contributing risk factors for preterm birth, yet its specific role remains largely unknown. Examining the exposure to stressors over a mother’s life course might provide more perspective on the role of maternal stress in preterm birth. Our aim was therefore to retrospectively explore the associations between chronic, lifelong stressors and protective factors and spontaneous preterm birth.

**Methods:**

This study was part of a large case–control study based in Edmonton, Canada, examining gene-environment interactions and preterm birth. Cases were mothers with a spontaneous singleton preterm birth (<37 weeks) without preterm premature rupture of membranes. Controls were mothers with an uncomplicated singleton term birth without a history of preterm birth. Sociodemographic and medical data were collected. A postpartum telephone questionnaire was administered to assess stressors across the lifespan. Both individual and contextual variables that could influence stress response systems were examined. Overall, 622 women were included, of which 223 subjects – 75 cases and 148 controls – completed the stress questionnaire. Univariate and multivariate logistic regression analyses were performed.

**Results:**

Multivariate analysis showed that exposure to two or more adverse childhood experiences (ACEs) was associated with a two-fold risk of preterm birth, regardless of maternal age, smoking status, educational status, and history of miscarriage (adjusted OR, 2.09; 95 % CI, 1.10–3.98; *P* = 0.024). The adjusted odds ratio for the ACE score was 1.18 (95 % CI, 0.99–1.40), suggesting that for every increase in childhood adverse event endorsed, the risk of preterm birth increased by 18 %. Lifetime physical and emotional abuse was also associated with spontaneous preterm birth in our study population (adjusted OR, 1.30; 95 % CI, 1.02–1.65; *P* = 0.033).

**Conclusions:**

A strong relationship between ACEs and preterm birth was observed. It has been shown that two or more ACEs have a notable two-fold increase in the risk of spontaneous preterm birth. These data demonstrate that stressors throughout life can have a significant effect on pregnancy outcomes such as preterm birth.

**Electronic supplementary material:**

The online version of this article (doi:10.1186/s12916-015-0353-0) contains supplementary material, which is available to authorized users.

## Background

With an estimated 15 million preterm births annually worldwide, the global burden of preterm birth (defined as birth before 37 completed weeks of gestation) is enormous [1]. Yet, its etiology remains largely elusive. Preterm birth is a complex phenomenon with genes and environmental factors contributing to its risk, both in the mother and the fetus. Further, maternal stress is increasingly recognized as a variable in the etiology of spontaneous preterm birth.

Significant antepartum and postpartum maternal stress can arise from multiple current circumstances or past sources across the lifespan; however, its specific contribution to the risk of preterm birth remains controversial. High levels of psychosocial stress experienced by women before or during pregnancy increase the risk of preterm labour [2–8]. Women who experienced major and traumatic life events early in pregnancy were also found to have an increased risk of preterm birth [9–11] although these were only associated with preterm birth when they were perceived to be stressful [12]. Indeed, women who have increased perceptions of stress also have a higher risk of preterm delivery [2, 6, 7]. Other stressors, including physical and emotional abuse or domestic violence prior to or during pregnancy [13–16], and distressed states, such as major depressive disorder and anxiety, also are associated with the onset of preterm labour [6, 17].

Low socio-economic status is also believed to be an important risk factor for preterm birth [18–20]. Socio-economic disadvantage is associated with unhealthy or risky behaviours, exposure to stress, and psychological reactions that influence gestation negatively [19]. Indeed, behavioural risk factors, such as cigarette smoking, alcohol and drug use, sexually transmitted infections, poor food intake, and obesity, are all associated with preterm birth [21–27].

Maternal stress and preterm birth share the elements that they are complex entities with many different environmental and psychosocial components. Joint examination of common stressors and individual socio-economic, psychosocial, and behavioural risk factors should therefore provide an effective strategy for increasing our understanding of the complex causes of preterm delivery [28–30]. In the past, studies examining the effect of maternal stress during pregnancy on preterm birth have had varied results, partly due to the fact that they only explored the relationship between separate stressors and preterm birth; often, cognitive appraisal of stressors or individual responses were not considered in the studies. Moreover, there is a lack of the use of a comprehensive measure of chronic stress. Examining the exposure to stressors over a mother’s life course might provide a better perspective on the role of maternal stress in the etiology of spontaneous preterm birth.

Chronic stress can lead to dysregulation of the neuroendocrine system resulting in acceleration of disease processes, an increase in inflammatory cells and cytokines and chronic activation of the inflammatory response [31–33]. Hence, the concept of allostatic load [34–38] – the wear and tear of the adaptive neuroendocrine systems in the body over a lifetime – provides a compelling rationale for the contribution of chronic stress to spontaneous preterm birth. In addition, creating a novel overall “stress index”, which takes into account both “stress load” and protective factors may allow further clarity regarding its relationship to preterm birth.

The objective of this study, therefore, was to retrospectively explore the associations between chronic, lifelong stressors and spontaneous preterm birth in our case–control study. We hypothesized that stress scores would be higher in women with preterm birth compared to controls, suggesting that higher levels of chronic maternal stress increase the risk of spontaneous preterm birth.

## Methods

### Study participants

This study was part of a case–control study based in Edmonton, Canada, examining both genetic variants and environmental factors in the etiology of spontaneous preterm birth. Cases were defined as mothers who gave birth to a singleton at less than 37 weeks gestation (preterm) as a result of spontaneous idiopathic preterm labour with intact membranes. Both vaginal delivery and caesarean sections were included as cases as long as the women with uncomplicated preterm caesarean sections began contracting spontaneously prior to 37 weeks. Controls were mothers with either a spontaneous, uncomplicated birth of a singleton at 38 to 41 weeks gestation or an (elective) uncomplicated caesarean section between 38 and 41 weeks gestation. Women with a delivery between 37 and 38 weeks were excluded to clearly delineate the groups due to the accepted error in dating gestation by last menstrual period or second trimester ultrasound (±7 days). Similarly, women with a history of preterm delivery in the control group were excluded. Other exclusion criteria included pre-eclampsia, placental abruption, uterine malformations, minor and major fetal malformations, HIV or AIDS, influenza, H1N1, and non-English speaking women. The Human Research Ethical Boards of the University of Alberta, Alberta Health Services and Covenant Health, approved the study. Between January 2009 and August 2010, women who delivered in one of the three Edmonton hospitals were approached and screened for eligibility. Written informed consent was obtained from all subjects before participating in the study.

### Collection of demographic data

Demographic and medical data were primarily collected from medical charts. It was not possible to obtain all desired data as medical records were often incomplete or certain demographic data were not recorded. Missing data were collected via self-report at enrolment and/or during follow-up telephone interviews.

Maternal medical charts were used to extract key medical variables and risk factors for preterm birth. Maternal data included maternal age, parity, height, and pre-pregnant weight for body mass index calculation, smoking, alcohol, and drug use. A history of uterine malformations, cervical procedures, medication use and pre-existing medical conditions, such as hypertension, diabetes mellitus, and autoimmune diseases, were also recorded. Additionally, information regarding mode of conception, method of gestational age determination, blood pressure (both early gestation and at term), cervical cerclage, and any medication use during pregnancy. We also extracted data concerning common complications during pregnancy, including genital tract infections and sexually transmitted infections, gestational hypertension or diabetes, polyhydramnios, and placental complications. Labour and delivery records were examined to determine gestational age at delivery, type of labour and mode of delivery, medication, evidence of maternal infection, and placental histopathology. Fetal data abstracted from the delivery records included sex of the infant, Apgar scores, cord pH, congenital malformations, and the evidence of infection in the first 48 hours.

Self-reported variables included height and pre-pregnancy weight, ethnicity, determinants of socio-economic status (marital status, neighbourhood, educational level, annual income of the household, and occupation), substance use, self-report medical and obstetric history, previous preterm births and/or miscarriages, and family history of preterm birth. Ethnicity was reported by self-identification back three generations from both the maternal and paternal side, where possible.

Data were subsequently stored in the online database and downloaded onto spreadsheets for analysis.

### Collection of stress data – the wellbeing and pregnancy questionnaire

For the assessment of chronic, lifelong stressors, we designed the ‘Well-being and Pregnancy Questionnaire.’ Using this questionnaire, both individual and contextual variables that influence the stress response were examined for all subjects. It incorporated several checklists designed for this study and validated research instruments to measure concepts related to stress and personal resources. They included perceived stress, common stressors during pregnancy, social support, life events, coping, adverse childhood experiences (ACEs), adult interpersonal violence experiences and depression. Instruments were chosen after review of the literature and based on their possible direct and/or indirect association with spontaneous preterm birth. Where possible, we used validated tools available in the public domain. In short, the questionnaire is comprised of the following tools: Perceived stress, Common stressors in pregnancy, Interpersonal Support Evaluation List (ISEL) [39], Life Events Checklist [40], Brief COPE [41], ACE Score [42], Abuse Assessment Screen (AAS) [43], and Mini International Neuropsychiatric Interview – modified sections A and C [44]. A more complete description of the Wellbeing and Pregnancy Questionnaire can be found in Additional file 1.

Between 3 months and 1 year postpartum, participating subjects were contacted by telephone where possible for follow-up and administration of the ‘Wellbeing and Pregnancy Questionnaire.’ To maximise the number of respondents, we attempted to contact each participant at least three times at different times during days and evenings. Answers were entered into our secure online database.

### Statistical analyses

All data were analyzed using SPSS 19.0 statistical software. Before analysis, data from different sources were merged and the data set was cleaned. Data were coded or recoded for analysis when required and missing data were indicated. Demographic and medical variables were compared between case and control subjects. For this univariate analysis, variables were compared using χ^2^ or binominal logistic regression, and odds ratios (OR) and 95 % confidence intervals (95 % CI) were recorded. A *P* value <0.05 was considered significant.

Scores for all questionnaire tools were separately calculated using predefined scoring keys. We also calculated a combined childhood and adult abuse score. For this score, the separate scores of childhood abuse, childhood neglect and adult physical and emotional abuse were added. In addition, a total combined stress score was computed, so that tools that represent stressors were added, while tools that represent modifiers of the stress response – social support and adaptive coping – were subtracted. This score was computed so that all stressors had the same weight. Univariate analysis was performed on all separate questionnaire tools and the total stress score to assess the relationship with spontaneous preterm birth. Some scores were also dichotomised based on their median split and subsequently analysed. Variables were compared using binominal logistic regression and ORs and 95 % CIs were recorded. A *P* value <0.05 was considered significant. Finally, multivariate logistic regression was performed. A multivariate model was created including the demographic variables that were significantly different between cases and controls in our population. Adjusted ORs and 95 % CIs were reported.

## Results

A total of 622 women were recruited to the study; 210 case group participants and 412 control group participants. In total, 234 telephone questionnaires were administered (37.6 % call rate). However, 11 study subjects that completed the questionnaire were later excluded from the study following secondary exclusion. Reasons for exclusion were uterine malformation (1 respondent), delivery between 37 and 38 gestational weeks (4 respondents), preterm premature rupture of membranes (1 respondent), placental abruption (1 respondent), history of preterm birth in control (1 respondent), and no spontaneous preterm labour (3 respondents). As a result, 223 completed telephone questionnaires were included in the study as responded by 148 controls and 75 cases. Our final call rate was 36 % for controls (148/412) and 36 % for cases (75/210).

### Univariate analysis

All socio-demographic and medical variables were compared between the case and control group (210 vs. 412 women) and their possible relationship with spontaneous preterm birth was assessed. Table 1 describes the main socio-demographic characteristics of our study population.

**Table 1:** Main demographic characteristics of the study population

Characteristic	Cases, n = 210	Controls, n = 412	OR^a^	95% CI	*P*
Maternal age, year^b^	28.3 ± 5.6	29.6 ± 5.2	0.96	0.93–0.99	0.004
Caucasian, n (%)	177 (84)	341 (83)	1.12	0.71–1.75	0.63
Smoking, n (%)	62 (30)	69 (17)	2.08	1.41–3.09	<0.001
Alcohol, n (%)	12 (6)	7 (2)	3.51	1.36–9.04	0.009
Street drugs, n (%)	15 (7)	8 (2)	3.89	1.12–9.32	0.002
Educational status					0.008^c^
High school diploma or less, n (% of known status)	34 (45)	37 (25)	Reference		
Undergraduate degree, n (% of known status)	35 (46)	99 (66)	0.39^d^	0.21–0.70	0.002
Graduate degree, n (% of known status)	7 (9)	14 (9)	0.54^d^	0.19–1.51	0.24
Marital status					0.43^c^
Pre-pregnant BMI^b^	26 ± 6.7	26 ± 6.2	1.00	0.97–1.03	0.93
Parity	0.78 ± 1	0.68 ± 0.89	1.12	0.93–1.33	0.21
Previous miscarriage, n (%)	68 (32)	96 (23)	1.58	1.09–2.28	0.015
ART, n (%)	6 (3%)	13 (3%)	0.79	0.30–2.09	0.63
Gestational age, wks^b^	33.7 ± 2.5	39.7 ± 1.0			<0.001
Birth weight, g^b^	2269 ± 584	3531 ± 461			<0.001

Variables were analyzed using χ^2^ test or univariate logistic regression. ^a^Odds ratio for spontaneous preterm birth; ^b^Mean ± standard deviation; ^c^Analyzed as continuous variable; ^d^Compared to reference group

Gestational age and birth weight were significantly different between cases and controls (*P* <0.001). On a continuous scale, maternal age was significantly inversely associated with spontaneous preterm birth (OR, 0.96; 95 % CI, 0.93–0.99). Overall, mothers in our case group were younger than controls (mean age 28.3 years vs. 29.6 years). Not surprisingly, substance use was also associated with spontaneous preterm birth. The ORs of smoking, alcohol use, and street drug use were 2.08 (1.41–3.09), 3.51 (1.36–9.04), and 3.89 (1.12–9.32), respectively. In addition, educational status had a significant relationship with preterm birth in our population. Of the women in the control group, 75 % completed education beyond high school, whereas only 55 % of the women in the case group completed undergraduate education. Other factors of socio-economic status, such as marital status and income, were not different between cases and controls, nor was ethnicity. Notably, a history of one or more miscarriages in previous pregnancies was significantly associated with preterm birth (OR, 1.58; 95 % CI, 1.09–2.28). None of the medical variables were significantly associated with spontaneous preterm birth in our study population.

Of all separate questionnaire instruments, only the ACE score was significantly associated with spontaneous preterm birth in univariate analyses (Table 2); the crude OR of ACE score on a continuous scale was 1.26 (95 % CI, 1.08–1.48). We also dichotomized the ACE score into high (≥2 ACEs) versus low ACE, based on median split, showing a crude OR on the risk of preterm birth of 2.45 (95 % CI, 1.37–4.38). Crude ORs for perceived stress, common stressors, ISEL, and COPE were all very close to 1, with ORs of 1.01 (95 % CI, 1.00–1.02), 1.09 (95 % CI, 0.92–1.30), 0.91 (95 % CI, 0.78–1.06), 1.04 (95 % CI, 0.91–1.20), and 1.02 (95 % CI, 0.97–1.06), respectively.

**Table 2:** Univariate analysis of all stress questionnaire tools and computed total stress score

Questionnaire tool	Crude OR	95% CI
Perceived stress	1.01	1.00–1.02
Common stressors	1.09	0.92–1.30
ISEL social support	0.91	0.78–1.06
Life events checklist	1.04	0.91–1.20
COPE adaptive coping	1.02	0.97–1.06
Adverse childhood experience (ACE)	1.26*	1.08–1.48
High ACE score (≥2 ACE)^a^	2.45*	1.37–4.38
Abuse assessment screen	1.75	0.96–3.20
Childhood and adult abuse	1.40*	1.13–1.74
Depression during pregnancy	1.53*	1.01–2.33
Lifetime history of depression	1.70	0.90–3.24
Total stress	1.46*	1.08–1.96
High stress^a^	1.86*	1.06–3.28

^a^ Based on median split; *P <0.05

Physical and emotional abuse as an adult, assessed with the AAS on its own, was not associated with preterm birth in our study. However, the combined abuse score of childhood and adult abuse was significantly associated with preterm birth (crude OR, 1.40; 95 % CI, 1.13–1.74). We found a significant relationship between the computed Total Stress score and spontaneous preterm birth after univariate logistic regression, showing a crude OR for the risk of preterm birth of 1.46 (95 % CI, 1.08–1.96). After dichotomization, a high stress score had an even greater crude OR of 1.86 (95 % CI, 1.06–3.33). The presence of depressive symptoms during pregnancy was significantly associated with preterm birth in our univariate analysis (crude OR, 15.3; 95 % CI, 1.01–2.33). A lifetime history of major depression had a fairly high crude OR of 1.70; however, this was not significant (95 % CI, 0.90–3.24).

**Figure 1:**
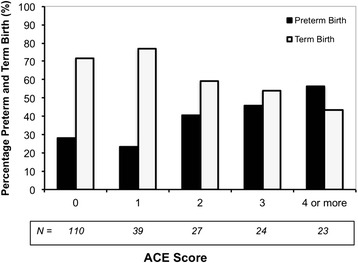
Relationship of Adverse Childhood Experience (ACE) score to preterm and term birth χ^2^ for linear trend *P* = 0.003

When examining more specifically the relationship between ACE score and spontaneous preterm birth, we found that the proportion of women with preterm birth gradually increased with increasing number of ACEs. Inversely, the percentage of women with a term birth decreased as the number of ACEs increased (Figure 1). The χ^2^ test for trend confirmed there was a linear trend (*P* = 0.003).

### Multivariate analysis

Univariate analyses of the socio-demographic and medical variables demonstrated that maternal age, smoking, alcohol and drug use, educational status, and a history of miscarriage were significantly associated with preterm birth in our study population. In our multivariate model, we therefore included maternal age, smoking, education, and history of miscarriage as covariates. We excluded alcohol use and drug use since the number of respondents reporting alcohol and/or drug use was very small.

The ACE score was almost significantly associated with spontaneous preterm birth after adjusting for maternal age, smoking, and educational status (Table 3). The adjusted OR for ACE score was 1.18 (95 % CI, 0.99–1.40), suggesting that, for every increase in childhood adverse event endorsed, the risk of preterm birth increased by 18 %. Notably, a high ACE score of two or more ACEs was associated with more than a two-fold increase in the risk of spontaneous preterm birth (adjusted OR, 2.09; 95 % CI, 1.10–3.98). When exploring the effect of lifetime abuse – combining childhood and adult abuse scores – we found that, with each additional increment of 1 on the abuse score scale, the risk of spontaneous preterm birth increased by 34 % (adjusted OR, 1.30; 95 % CI, 1.02–1.65). Although the ORs of total stress score and high stress score for preterm birth remained high in our multivariate model, neither total stress nor a high stress score was significantly associated with spontaneous preterm birth (adjusted OR, 1.26; 95 % CI, 0.90–1.76 and adjusted OR, 1.61; 95 % CI, 0.88–2.94, respectively). The same was true for the presence of depressive symptoms during pregnancy (adjusted OR, 1.42; 95 % CI, 0.91–2.22).

**Table 3:** Multivariate analyses of total stress, Adverse Childhood Experience (ACE) score, lifetime abuse, and depression

Questionnaire tool	Adjusted odds ratio^a^	95% CI
Total stress	1.26	0.90–1.76
High stress^b^	1.61	0.88–2.94
ACE score	1.18	0.99–1.40
High ACE score (≥2 ACEs)^b^	2.09*	1.10–3.98
Childhood and adult abuse	1.30*	1.02–1.65
Depression during pregnancy	1.42	0.91–2.22

^a^Adjusted for maternal age, educational status, smoking and history of miscarriage; ^b^Based on median split; *P <0.05

## Discussion

This study demonstrates that there is a strong relationship between ACEs – assessed with the ACE survey – and spontaneous preterm birth in later life. Every additional ACE increased the risk of spontaneous preterm birth by 18 %. This was after adjustment for maternal age, smoking, educational status, and history of miscarriage, all of which were found to be confounding factors in our study. More importantly, being exposed to two or more ACEs prior to the age of 18 was associated with a highly significant two-fold increase in the risk of delivering an infant preterm. Given the baseline risk of spontaneous preterm birth of around 9 % to 10 % [1], this meant that having experienced two or more ACEs during childhood increased the risk of preterm birth to 20 %, regardless of age, smoking, educational status, or history of miscarriage. In addition, our study showed that, with increasing number of ACEs, the proportion of women with a term birth decreased, whereas the proportion of women with a preterm birth increased (Figure 1).

We also found that ACEs were more prevalent in cases than controls (data not shown). For instance, 18 % of the women in our case group experienced sexual abuse as a child compared to 8 % of the control women. A similar difference was seen in the prevalence of emotional neglect: 20 % and 6 % of the women were emotionally neglected during childhood in the case and control groups, respectively. Apart from criminal behaviour, i.e., a household member imprisoned, all ACEs regarding household dysfunction were very common in both groups of women, occurring in up to 23 % of cases. In addition, almost a quarter of all women with a preterm birth admitted to being physically abused during childhood, while 15 % of the controls experienced physical abuse as a child.

ACEs have been found to be associated with a large diversity of long-term negative health outcomes and risky behaviour [42], including depression [45], ischemic heart disease [46], obesity [47], fetal death [48], sexually transmitted infections [49], alcohol abuse [50, 51], smoking [52, 53], drug use [51], and adolescent pregnancy [48]. Many of these adverse health outcomes and health risk behaviours associated with ACEs are directly associated with preterm birth as well. Smoking, alcohol use, obesity, adolescent pregnancy, and depression have all been found to be associated with both preterm birth and ACEs. Indeed, several of these factors were significantly associated with spontaneous preterm birth in our study population. It is very likely that adverse experiences in childhood interact with the various socio-demographic and medical risk factors for preterm birth resulting in increased risks of preterm birth. Our study sample was, however, not adequately powered to test for these possible interactions. Adult abuse on its own was not associated with spontaneous preterm birth. Yet, when we combined the scores of childhood abuse and neglect from the ACE score and adult abuse as defined by the AAS, we discovered a significant relationship between lifetime abuse and preterm birth. We found that, with each additional increment of 1 on the abuse score scale, the risk of spontaneous preterm birth increased by 30 %. That is much higher than the 18 % increase of risk found with each additional increment on the ACE score. These data propose that, when measures of childhood and adult abuse are taken together, a nearly synergistic effect was seen for the risk of spontaneous preterm birth.

Evidence suggests that ACEs can lead to hyper-reactivity of the hypothalamic-pituitary-adrenal and sympatho-adrenal-medullary axes in response to stress in adulthood [54]. This effect is even stronger in women with symptoms of depression. It is believed that ACEs can induce persistent changes in the systems involved in the stress response leading to many negative health outcomes, including depression [42, 55]. This is consistent with the concept of allostatic load [34, 35]; in chronic stress, the allostatic load increases as the body attempts to cope with stressors. Over a long period of time, this might then cause the allostatic system exhaustion, leading to dysregulation of the hypothalamic-pituitary-adrenal axis and compensatory responses in other systems. Chronic stress can therefore result in an increase in inflammatory cells and cytokines and increased susceptibility to infection and inflammation. Thus, it is biologically very plausible that chronic stress, and more specifically, ACEs, can increase the risk of preterm birth via the neuroendocrine and inflammatory pathways. We believe, therefore, that a healthy pregnancy starts long before conception.

### Study strengths and limitations

Our telephone survey response rate was surprisingly high, at 37.6 %, given response rates for telephone surveys have been steadily declining over two decades [56]. Nevertheless, compelling evidence exists that response rate is not necessarily an indicator of survey quality. Two research teams using very different experimental designs found little evidence for a relationship between response rate and non-response bias in telephone surveys [57, 58]. Groves [59] noted that the collective body of empirical work suggests no consistent relationship between response rates and non-response bias.

A concern for every retrospective study is the issue of recall bias. Fortunately, research indicates that the number of non-responders has little or no effect on the validity of the data obtained from the responders [58–60]. Regardless, we took steps to minimize its impact. For instance, all the instruments used in the questionnaire contained only questions about lifetime events and how women feel and respond in general and at specific time points in life. The majority of instruments incorporated in the questionnaire, such as the ACE score, were designed and validated for retrospective assessment and therefore suitable for our study design. Post-partum depression was a concern, but it affects mothers (and fathers) of term as well as preterm newborns. To minimize its effects, we delayed initiating any contacts with subjects until 3 months post-partum. Importantly, our Pregnancy and Well-being Questionnaire, comprised of eight separate instruments, made use of re-survey, which minimizes recall bias. It is likely that off-setting biases also minimized recall biases. Finally, we had exactly the same proportion of case–control responders (1:2) as we did in the overall study.

We decided that, for our purpose, the preferred method of stress assessment was via telephone as there were no convenient or routine clinic appointment times and the response rate from a mailed questionnaire would be too low. In addition, some of the questions in the questionnaire might be upsetting and require direct contact with the study coordinator. The time for the telephone questionnaire was kept to less than 30 minutes; this limited the number of instruments that could be used. Our telephone response rate was 37.6 %, which is well above average for such telephone questionnaires. There were no significant differences in the characteristics of the populations who responded to the telephone interview from the characteristics of the overall group recruited.

Our population was mostly Caucasian (84 % and 83 % in cases and controls, respectively) with an insignificant contribution from other races. There were no differences in ethnic background (Caucasian, Asian, Black, Hispanic, and Aboriginal) between the case and control groups. Hence, the association between ACEs and preterm birth observed was not due to race. After adjusting for confounding variables, a high total stress score – comprising all measures of chronic stress – was not associated with spontaneous preterm birth. One explanation for this could be the small sample size, but it could also be explained by the method of calculating this score. No composite measure of chronic stress, including perceived stress, common stressors, social support, life events, coping, ACEs, adult abuse, and depression exists in the literature nor does any standardized calculation of chronic stress. The development of better and, most importantly, standardized composite measures of chronic stress will aid in the assessment of chronic stress and the overall role of allostatic load in preterm delivery.

## Conclusions

In summary, ACEs are associated with spontaneous preterm birth. After adjustment for confounding variables, we found that women who were exposed to two or more ACEs have a notable two-fold increase in the risk of preterm birth. In addition, lifetime abuse was also linked to preterm birth. The data demonstrate that stressors throughout life can have a significant effect on pregnancy outcomes, including preterm birth. A healthy pregnancy therefore starts long before conception.

## Additional file

Additional file 1:
**The Wellbeing and pregnancy questionnaire.**

## Abbreviations

AAS: Abuse Assessment Screen
ACE: Adverse childhood experience
CI: Confidence interval
ISEL: Interpersonal Support Evaluation List
OR: Odds ratio
