# Supplementary material for: Adverse childhood experiences are associated with spontaneous preterm birth: a case–control study
Source: BMC Med. 2015 Jun 11;13:124. doi: 10.1186/s12916-015-0353-0 (PMC4464612; doi:10.1186/s12916-015-0353-0)
Supplement: Additional file 1: — The Wellbeing and pregnancy questionnaire. [file 12916_2015_353_MOESM1_ESM.docx]

# Supplemental Material

## The wellbeing and pregnancy questionnaire

We designed the ‘Well-being and Pregnancy Questionnaire for the assessment of chronic, lifelong stressors. Both individual and contextual variables that influence the stress response were examined using this questionnaire. It incorporated several checklists designed for this study and validated research instruments to measure concepts related to stress and personal resources. Where possible, we used validated tools that are available in the public domain. The instruments that were incorporated in the questionnaire are described below.

### Perceived stress

The first instrument measured a global level of perceived stress before and during pregnancy. Women were asked to indicate how stressed they felt, on a scale from zero to one hundred, six months prior to their pregnancy – as a reference score – in their first trimester and in their second trimester. This instrument was designed for this study and was loosely based on the validated Perceived Stress Scale [[1](#_ENREF_1)].

### Common stressors in pregnancy

The second instrument was a checklist designed to assess the presence of common stressors during pregnancy and intended to measure stress load during pregnancy. Common stressors assessed included high workload, financial problems, personal conflicts at home and at work, parenting problems, perceived racial discrimination and unfavourable neighbourhood.

### Interpersonal Support Evaluation List

Strong support networks are associated with a person’s well-being [[2](#_ENREF_2)]. To assess social support, we used the validated Interpersonal Support Evaluation List (ISEL) – Short Form. This is a 15-item measure of perceived social support [[3](#_ENREF_3)]. This short version was derived from the 48-item ISEL, designed by Cohen [[4](#_ENREF_4)]. The three areas assessed were tangible support or material aid, appraisal support or the availability of a confidant, and belonging support or the availability of someone with whom the respondent can socialize or relax. In a study in 2000, Widows *et al*. found adequate internal consistency of the ISEL [[5](#_ENREF_5)].

### Life Events Checklist

The fourth instrument, which assessed historical exposure to stressors, was the Life Events Checklist (Page 1) of the Clinician Administered Posttraumatic Stress Disorder Scale (CAPS 1) (National Center for Posttraumatic Stress Disorder) [[6](#_ENREF_6), [7](#_ENREF_7)]. This scale was originally developed by the National Center for Posttraumatic Stress Disorder as part of a diagnostic interview to assess presence of posttraumatic stress disorder diagnostic status and symptom severity. Listed were several stressful life events that have all been shown to increase risk of stress-related disorders. In a review, Weathers *et al*. found excellent reliability rating and convergent and discriminant validity were also shown to be strong [[8](#_ENREF_8)].

### Brief COPE

Adaptive coping styles are also associated with well-being. The Brief COPE is a 28-item measure shortened from the original 60-item COPE [[9](#_ENREF_9), [10](#_ENREF_10)]. It was used to assess situational reports of coping as well as dispositional coping styles. The different coping strategies assessed were passive/avoidant behaviour, action oriented behaviour, and emotional coping. The Brief COPE scales all met the criteria for internal reliability [[9](#_ENREF_9)].

### Adverse Childhood Experiences Score

Our sixth tool was the Adverse Childhood Experiences (ACE) Score. This questionnaire investigated the connection between adult health problems, including preterm birth, and adverse childhood experiences. It has long been known that significant life experiences can affect health in later life. Childhood abuse, neglect and dysfunctional household are associated with increased health risks, such as smoking, alcoholism and drug abuse, and many adult diseases, such as cardiovascular disease and depression [[11](#_ENREF_11)]. The ACE Score is a 10-item measure to identify childhood abuse, neglect and household dysfunction - all questions pertained to the respondent’s first 18 years of life. The test-retest reliability was found to be in the good range with kappa coefficients between 0.6 and 0.7 [[12](#_ENREF_12)].

### Abuse Assessment Screen

The instrument used for the assessment of abuse as an adult was the Abuse Assessment Screen (AAS) [[13](#_ENREF_13)]. The AAS has been widely used to identify abuse during pregnancy in health settings. Women were asked questions about emotional and physical abuse during life and during their pregnancy. Content and criterion validity and test-retest reliability have been established [[14](#_ENREF_14), [15](#_ENREF_15)].

### Depression and suicidality

Depression is a contributor to the stress response. It can activate the stress response directly and it can affect a person’s cognitive appraisal of stressors [[16](#_ENREF_16)]. In addition, it can also be an outcome of chronic stress as high levels of stressors can cause the development of depression and anxiety. Depression prior to and during pregnancy is associated with preterm birth [[17](#_ENREF_17), [18](#_ENREF_18)]. For the assessment of depression and suicidality, we used sections A and C of the validated Mini International Neuropsychiatric Interview (M.I.N.I.) [[19](#_ENREF_19)]. We slightly modified the scales for our questionnaire and both sections include questions about depressive episodes and thoughts of suicidality both before and during pregnancy. In addition, the presence of post-partum depression was assessed

## References

1. Cohen S, Kamarck T, Mermelstein R. A global measure of perceived stress. Journal of health and social behavior. 1983;24(4):385-96.

2. Cohen S, Wills TA. Stress, social support, and the buffering hypothesis. Psychological bulletin. 1985;98(2):310-57.

3. Peirce RS, Frone MR, Russell M, Cooper ML. Financial stress, social support, and alcohol involvement: a longitudinal test of the buffering hypothesis in a general population survey. Health psychology : official journal of the Division of Health Psychology, American Psychological Association. 1996;15(1):38-47.

4. Cohen S, Mermelstein R, Kamarck T, Hoberman HM. Measuring the functional components of social support. In: Sarason I, Sarason B, editors. Social support: theory, research, and applications. Boston: Martinus Nijhoff; 1985. p. 73-94.

5. Widows MR, Jacobsen PB, Fields KK. Relation of psychological vulnerability factors to posttraumatic stress disorder symptomatology in bone marrow transplant recipients. Psychosomatic medicine. 2000;62(6):873-82.

6. Blake DD, Weathers FW, Nagy LM, Kaloupek DG, Gusman FD, Charney DS et al. The development of a Clinician-Administered PTSD Scale. Journal of traumatic stress. 1995;8(1):75-90.

7. Gray MJ, Litz BT, Hsu JL, Lombardo TW. Psychometric properties of the life events checklist. Assessment. 2004;11(4):330-41. doi:10.1177/1073191104269954.

8. Weathers FW, Keane TM, Davidson JR. Clinician-administered PTSD scale: a review of the first ten years of research. Depression and anxiety. 2001;13(3):132-56.

9. Carver CS. You want to measure coping but your protocol's too long: consider the brief COPE. International journal of behavioral medicine. 1997;4(1):92-100. doi:10.1207/s15327558ijbm0401_6.

10. Carver CS, Scheier MF, Weintraub JK. Assessing coping strategies: a theoretically based approach. Journal of personality and social psychology. 1989;56(2):267-83.

11. Felitti VJ, Anda RF, Nordenberg D, Williamson DF, Spitz AM, Edwards V et al. Relationship of childhood abuse and household dysfunction to many of the leading causes of death in adults. The Adverse Childhood Experiences (ACE) Study. American journal of preventive medicine. 1998;14(4):245-58.

12. Dube SR, Williamson DF, Thompson T, Felitti VJ, Anda RF. Assessing the reliability of retrospective reports of adverse childhood experiences among adult HMO members attending a primary care clinic. Child abuse & neglect. 2004;28(7):729-37. doi:10.1016/j.chiabu.2003.08.009.

13. McFarlane J, Parker B, Soeken K, Bullock L. Assessing for abuse during pregnancy. Severity and frequency of injuries and associated entry into prenatal care. Jama. 1992;267(23):3176-8.

14. McFarlane J, Parker B, Soeken K, Silva C, Reel S. Safety behaviors of abused women after an intervention during pregnancy. Journal of obstetric, gynecologic, and neonatal nursing : JOGNN / NAACOG. 1998;27(1):64-9.

15. Parker B, McFarlane J, Soeken K. Abuse during pregnancy: effects on maternal complications and birth weight in adult and teenage women. Obstetrics and gynecology. 1994;84(3):323-8.

16. Chrousos GP. Stressors, stress, and neuroendocrine integration of the adaptive response. The 1997 Hans Selye Memorial Lecture. Annals of the New York Academy of Sciences. 1998;851:311-35.

17. Dayan J, Creveuil C, Herlicoviez M, Herbel C, Baranger E, Savoye C et al. Role of anxiety and depression in the onset of spontaneous preterm labor. American journal of epidemiology. 2002;155(4):293-301.

18. Orr ST, James SA, Blackmore Prince C. Maternal prenatal depressive symptoms and spontaneous preterm births among African-American women in Baltimore, Maryland. American journal of epidemiology. 2002;156(9):797-802.

19. Sheehan DV, Lecrubier Y, Sheehan KH, Amorim P, Janavs J, Weiller E et al. The Mini-International Neuropsychiatric Interview (M.I.N.I.): the development and validation of a structured diagnostic psychiatric interview for DSM-IV and ICD-10. The Journal of clinical psychiatry. 1998;59 Suppl 20:22-33;quiz 4-57.
